# Keratinocyte Detachment-Differentiation Connection Revisited, *or Anoikis-Pityriasi Nexus Redux*

**DOI:** 10.1371/journal.pone.0100279

**Published:** 2014-06-24

**Authors:** Tomohiro Banno, Miroslav Blumenberg

**Affiliations:** 1 The R.O.Perelman Department of Dermatology, NYU Langone Medical Center, New York, New York, United States of America; 2 Department of Biochemistry and Molecular Pharmacology, NYU Langone Medical Center, New York, New York, United States of America; 3 NYU Cancer Institute, NYU Langone Medical Center, New York, New York, United States of America; CNRS-University of Toulouse, France

## Abstract

Epidermis, a continuously self-renewing and differentiating organ, produces a protective stratum corneum that shields us from external chemical, physical and microbial threats. Epidermal differentiation is a multi-step process regulated by influences, some unknown, others insufficiently explored. Detachment of keratinocytes from the basement membrane is one such pro-differentiation stimulus. Here, we define the transcriptional changes during differentiation, especially those caused by detachment from the substratum. Using comprehensive transcriptional profiling, we revisited the effects of detachment as a differentiation signal to keratinocytes. We identified the genes regulated by detachment, the corresponding ontological categories and, using metaanalysis, compared the genes and categories to those regulated by other pro-differentiating stimuli. We identified 762 genes overexpressed in suspended keratinocyte, including known and novel differentiation markers, and 1427 in attached cells, including basal layer markers. Detachment induced epidermis development, cornification and desmosomal genes, but also innate immunity, proliferation inhibitors, transcription regulators and MAPKs; conversely the attached cells overexpressed cell cycle, anchoring, motility, splicing and mitochondrial genes, and both positive and negative regulators of apoptosis. Metaanalysis identified which detachment-regulated categories overlap with those induced by suprabasal location *in vivo*, by reaching confluency *in vitro*, and by inhibition of JUN kinases. Attached and *in vivo* basal cells shared overexpression of mitochondrial components. Interestingly, melanosome trafficking components were also overexpressed in the attached and *in vivo* basal keratinocytes. These results suggest that specific pro-differentiation signals induce specific features of the keratinization process, which are *in vivo* orchestrated into harmonious epidermal homeostasis.

## Introduction

Human epidermis is a constantly self-renewing and differentiating structure, composed mainly of keratinocytes that proliferate in the basal layer and, after detachment, progress through a complex process that results in a protective stratum corneum. Disruption of epidermal differentiation results in severe diseases, from skin cancers and bullous diseases to non-healing wounds, inflammation and infections; this currently presents grave health issues [1]. Aspects of epidermal differentiation have been reviewed recently [2], [3].

The commencement of differentiation is regulated at several levels and by multiple influences, including Ca^++^ gradient, UV exposure, drug reactions etc. [4]–[7]. *In vitro*, keratinocyte differentiation can be induced by confluency, raising Ca^++^ levels, inhibition of JNK, Ephrins and other agents [4], [8]–[10]. In a pioneering study a decade-and-half ago Dr. Watt and her collaborators suggested that the detachment from the basement membrane is one of stimuli for differentiation; they showed that keratinocytes, prevented from attachment to a substratum, produce involucrin a canonical differentiation marker [11]. They also demonstrated that fibronectin can partially delay this process, and so can laminin and collagen [12]. These components of the basement membrane are all ligands for integrins, which suggested that integrin signaling, or rather lack thereof, plays a role in initiating keratinocyte differentiation [13], [14].

Our objective in this work was to define comprehensively the transcriptional changes in keratinocytes prevented from attachment to the substratum. This is important because several genodermatoses, such as epidermolysis bullosa, and blistering diseases, such as bullous pemphigoid involve keratinocyte detachment from the basement membrane [15], [16]. In these conditions, the epidermal differentiation is altered as a direct or indirect consequence of keratinocyte detachment.

We also wanted to compare the transcriptional profile of suspended keratinocytes to profiles of keratinocytes induced to differentiate by other means, namely in the suprabasal layers of the epidermis *in vivo*, keratinocytes grown to confluency in culture, and those treated with JNK inhibitors [10], [17]. Therefore, we prepared two batches of keratinocytes, one grown in standard tissue culture dishes, i.e., substrate-bound, the other in bacteriological Petri dishes, unable to attach, i.e., in suspension. We harvested the RNA from the two cultures and compared the transcriptomes using microarrays.

We found that detached keratinocytes indeed commence a complex differentiation program, which incorporates production of canonical differentiation markers, including components of the cornified envelopes. Simultaneously, the detached keratinocytes discontinue production of basal-layer characteristic proteins, including integrins and, notably, the cell cycle machinery. Suspension induces inhibitors of proliferation and enzymes of lipid and steroid metabolism, while suppressing mitosis, mitochondrial and attachment proteins, splicing and ribosomal components.

We then compared the transcription profile of suspended cells with the profiles of suprabasal epidermis [18], keratinocytes grown to confluency [17], and those treated with JNK inhibitor SP600125 [10]. Some of the differentiation markers and processes detachment shares with confluency (e.g., lipid metabolism), others with JNK inhibition (e.g., steroid metabolism), yet others are shared only with *in vivo* suprabasal cells (e.g., mitochondrial proteins), but not with other differentiation inducers. Overall, the results suggest that there are multiple, parallel and independent pathways leading to epidermal differentiation and that detachment from the substratum initiates a characteristic subset of these pathways.

## Results

**Figure 1:**
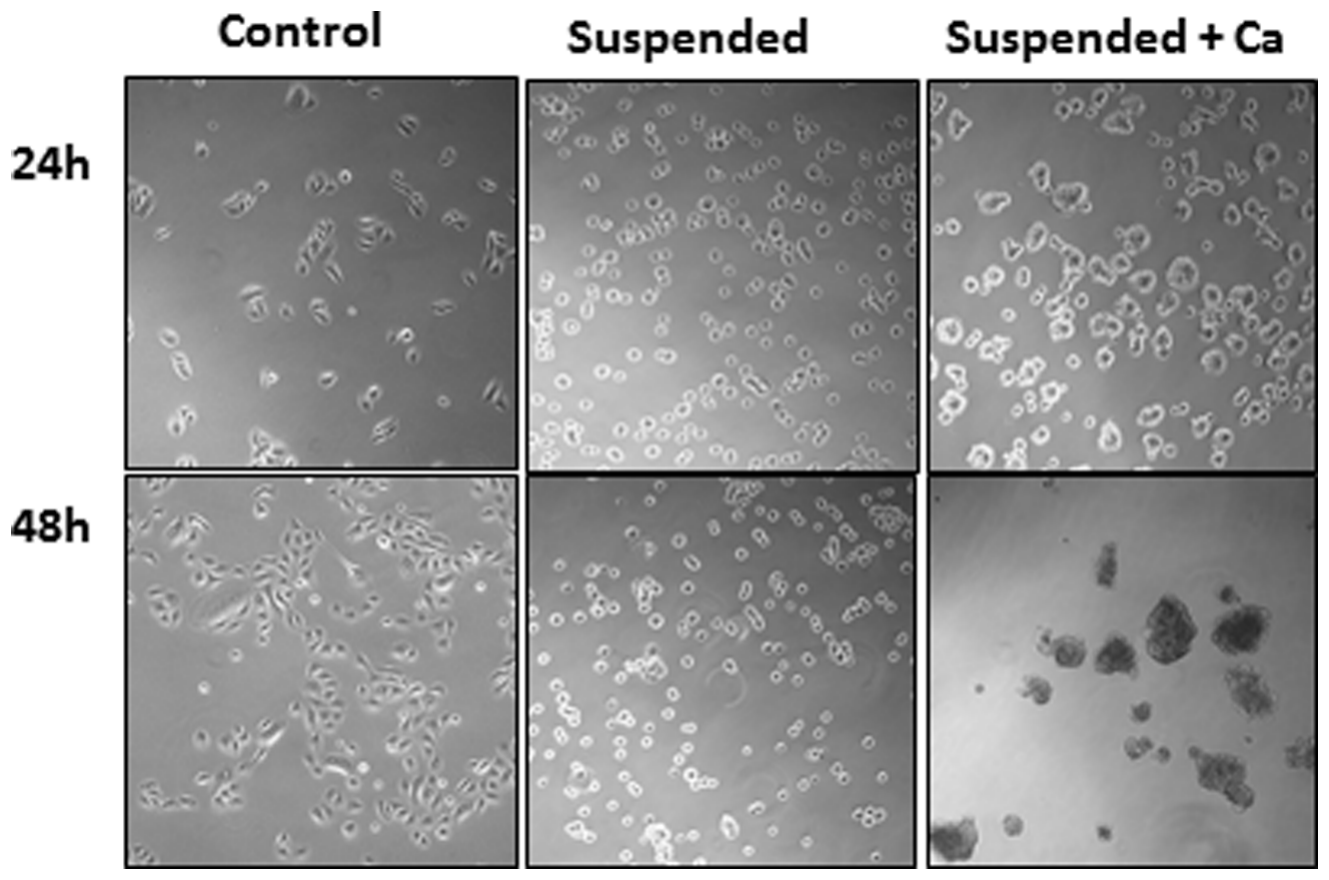
Suspended and attached keratinocytes. Cultures 24 and 48

To determine the molecular effects of detachment of keratinocytes from their substratum and the role detachment plays in initiating differentiation, we compared the transcriptomes of attached and suspended keratinocyte cultures. Keratinocytes in standard keratinocyte growth medium but in bacteriological culture plates remain alive but do not proliferate (Fig 1). They round up and remain suspended as single cells in the absence of Ca^++^; however, upon addition of Ca^++^ keratinocytes bunch up, connect to one another forming clumps that resemble dandruff (in Greek *pityriasi*, *πιτψριασι*). This suggests that in suspension keratinocytes produce junctional proteins, including desmosomes, markers of suprabasal, differentiating epidermal layers. The expression of desmosomal proteins supports the suggestion that keratinocytes in suspension commence differentiation [19].

We used microarrays to compare the transcription profiles of the attached and suspended keratinocytes. We compared a single line of primary cultured cells grown on tissue culture plates with the same cells grown in suspension, in bacteriological plates. We found 762 genes overexpressed in the suspended, and 1427 in the attached cultures; (genes were considered regulated if the expression levels differed more than 1.5-fold. The full lists of differentially expressed genes are given in the Supplement, Tables S1 and S2, resp. We confirmed the regulation of a subset of genes, specifically Transglutaminase 1 and Notch 1, using Northern blots (not shown).

### Genes induced in keratinocytes in suspension

If detachment initiates aspects of differentiation, we would expect the known keratinization markers to be expressed in suspended cultures. Indeed, the most prominent ontological category induced in suspended culture was ‘Epidermis development/Ectoderm development’ with p-value 2.53E-16 (Table 1a). This category includes such genes as involucrin, filaggrin, transglutaminase 1 and SPRRs, the canonical markers of epidermal differentiation; these are marked with double asterisks in Table 1a. Many of these are components of cornified envelopes (marked with asterisks in Table 1a). Moreover, the expression of desmosomal components and desmosomes-associated proteins was also prominently induced in suspension, p-value 4.37E-8 (Table 1b), corroborating the results shown in Fig. 1. These findings fully validate the suggestion that detachment of keratinocytes induced their differentiation.

**Table 1:** Ontological categories overexpressed in attached keratinocytes.

**a) Ectoderm development**	**c) Response to bacteria**
arachidonate 12-lipoxygenase, 12R type	adrenomedullin
BARX homeobox 2	CD24 molecule CD24 molecule-like 4
cellular retinoic acid binding protein 2	cytochrome P450, polypeptide 1
corneodesmosin **	defensin, beta 1 ∼∼
cystatin A (stefin A) **	defensin, beta 4 ∼∼
cystatin E/M	histone cluster 1, H2bi
desmoplakin **	histone cluster 1, H2bj
E74-like factor 5 (ets domain transcription factor)	histone cluster 1, H2bk
envoplakin **	histone cluster 2, H2be
epiregulin **	interleukin 6 receptor
fatty acid binding protein 5 (psoriasis-associated)	jun oncogene
filaggrin **	mitogen-activated protein kinase 14
forkhead box N1 **	NFkB inhibitor, alpha
involucrin **	protein phosphatase 1D, delta isoform
kallikrein-related peptidase 7	ribosomal protein S6 kinase, 70kDa, polypeptide 1
keratin 1 **	S100 calcium binding protein A12
keratin 10 **	S100 calcium binding protein A7
keratin 13	thrombomodulin
keratin 16	tribbles homolog 1
keratin 17	v-fos FBJ oncogene homolog
keratin 2 **	Wiskott-Aldrich syndrome-like
keratin 31	
keratin 9 **	**d) Negative regulators of proliferation**
Kruppel-like factor 4	adrenomedullin
late cornified envelope 2B **	alpha-2-glycoprotein 1
loricrin **	B-cell CLL/lymphoma 6
MAX interactor 1 **	B-cell translocation gene 1, anti-proliferative
ovo-like 1	bone morphogenetic protein 2
periplakin **	BTG family, member 2
peroxisome proliferator-activated receptor delta **	C/EBPalpha
S100 calcium binding protein A7 **	CD24 molecule
sciellin **	CDK inhibitor 1B (p27, Kip1)
serine peptidase inhibitor, Kazal type 5	CDK inhibitor 1C (p57, Kip2)
SMAD specific E3 ubiquitin protein ligase 1	formin binding protein 1
small proline-rich protein 1A **	gamma-glutamyl hydrolase
small proline-rich protein 1B (cornifin) **	glucosidase, beta acid
small proline-rich protein 2G **	hyaluronoglucosaminidase 1
small proline-rich protein 2D **	iduronate 2-sulfatase
thioredoxin interacting protein **	palmitoyl-protein thioesterase 2
TIMP metallopeptidase inhibitor 2 **	potassium voltage-gated channel 1
transducer of ERBB2, 1 **	sphingomyelin phosphodiesterase 1,
transglutaminase 1 (K epidermal type I) **	tripartite motif-containing 23
transglutaminase 3 (E polypeptide) **	ubiquitin specific peptidase 4 (proto-oncogene)
transglutaminase 5	ubiquitin specific peptidase 6 (Tre-2 oncogene)
transmembrane protein 115 **	
tribbles homolog 1 (Drosophila) **	**e) Response to steroid hormone**
UDP-glucose ceramide glucosyltransferase	adrenomedullin
v-erb-b2 oncogene homolog 2	carbonic anhydrase II
zinc finger and BTB domain containing 17	CD24 molecule
	crystallin, alpha B
**b) Desmosome**	cyclin E1
corneodesmosin	dual specificity phosphatase 1
desmocollin 1	E1A binding protein p300
desmocollin 2	GATA binding protein 3
desmoglein 1	heme oxygenase (decycling) 1
desmoplakin	interleukin 1 receptor antagonist
envoplakin	interleukin 6 receptor
junction plakoglobin	jun B proto-oncogene
periplakin	PPAR gamma
plakophilin 1	phosphatase and tensin homolog
plakophilin 3	protease, serine, 8
CDK4 inhibitor 2B (p15)	regulator of calcineurin 1
epiregulin	retinoid X receptor, alpha
heme oxygenase (decycling) 1	ribosomal protein S6 kinase, 70 kDa, polypeptide 1
hyaluronoglucosaminidase 1	thioredoxin interacting protein
insulin-like growth factor binding protein 5	v-erb-b2 oncogene homolog 2
jumonji, AT rich interactive domain 2	v-fos FBJ oncogene homolog
K(lysine) acetyltransferase 2B	
keratin 4	**f) MAPK Pathway**
Kruppel-like factor 10	C/EBPalpha
Kruppel-like factor 4	MAP kinase interacting serine/threonine kinase 2
vacuolar protein sorting 4 homolog B	mitogen-activated protein kinase 3
	jun oncogene
	mitogen-activated protein kinase 13
	mitogen-activated protein kinase 14
	mitogen-activated protein kinase 6
	mitogen-activated protein kinase 7
	mitogen-activated protein kinase kinase 3
	mitogen-activated protein kinase kinase kinase 4

a) Ectoderm development. Known components of the cornified envelopes are marked with double asterisks. b) Desmosomal; proteins. Some of these are also noted as ectoderm development markers. c) Response to bacteria; the two defensins are marked with tildes. d) Negative regulators of proliferation. e) Genes responding to steroid hormone regulation. f) Components of the MAPK signaling cascades.

One important function of differentiating keratinocytes is to provide an antimicrobial barrier. Indeed, we find that suspension induced the ‘Response to bacteria’ ontological category, p-value 1.09E-3 (Table 1c). Defensins beta-1 and beta-4 are among the induced genes. This suggests that innate immunity is an intrinsic aspect of keratinocyte differentiation.

Mitotic figures are only seen in the basal layer of the epidermis [20]. As keratinocytes leave the basal layer to commence differentiation, they simultaneously cease proliferation; this process involves induction of negative regulators of proliferation, with p-value 7.18E-5 (as well as suppression of the cell cycle machinery, see below). The negative regulators of proliferation induced in suspension are listed in Table 1d.

The physical barrier of the stratum corneum contains lipids, 50% ceramides, 25% cholesterol, and 15% free fatty acids and others [2]. Indeed, we find enzymes of fatty acid and cholesterol metabolism induced in suspended cultures (see below). However, we were surprised that ‘Response to steroid hormone’ is also a category induced by suspension, with p-value 1.03E-3 (Table 1e). The genes responsive to steroid hormone may be more associated with the innate immunity or the control of inflammation; adrenomedullin, a vasodilator, DUSP1, IL-1Ra and IL-6R may have such functions. This result also substantiates the findings that epidermis is a proximal source of steroid hormones and the ‘response to steroid hormone’ may be a response to the steroids endogenously produced in the epidermis [21], [22]. Importantly, steroid metabolism is also an important and well known aspect of epidermal differentiation [2]; therefore, the ‘Response to steroid hormone’ category may be directly related to the lipid metabolism aspect of epidermal differentiation.

Several genes encoding components of the MAPK pathway, including 5 MAPKs, are induced in suspension, p-value 1.23E-6 (Table 1f). Their role in epidermal differentiation has been noted in many studies [23], [24] and thus is confirmed here. The MAPK pathway activates the AP1 transcription factor, comprising Jun and Fos proteins, which are known to play significant roles in the expression of differentiation-specific keratin proteins [25], [26].

Importantly, the suspension of keratinocytes results in extensive changes of the transcription machinery (Table S3). Many positive as well as negative regulators of transcription are induced by suspension; depending on the context, many induced transcription regulators can act positively and negatively and, therefore, appear in both lists in Table S3 with p-values 5.95E-10 and 1.47E-6, resp. We note prominent among transcription factors in the positive regulators category are the members of the nuclear receptor family, RAR, RXR, T3R, PPARs and RAR-related orphan receptor A, and the associated nuclear receptor coactivators 1 and 2. These reflect the significant roles nuclear receptors play in regulating epidermal differentiation [27]–[29]. Interestingly, several viral oncogene homologs, v-ets, v-fos, v-maf and v-rel, are induced in suspended keratinocytes, as if the commencement of differentiation permits keratinocytes to express these genes, which may be too dangerous in basal, proliferation-competent cells.

### Genes overexpressed in the substrate-attached keratinocytes, compared to in the detached ones

One of the most prominent ontological categories differentially expressed in the attached keratinocytes is the cell cycle, with p-value 9.12E-19 (Table S4). Fully 150 genes belong to this category and include the CDC family, CDKs, cyclins, proteasome subunits, aurora kinases, kinesins and other microtubule associated cytoskeletal components, septins, as well as DNA replication proteins, e.g., MCM complex. The upstream regulators of cell cycle, such as EGFR, MAPKK1, RB1 etc., are also prominent in the attached keratinocytes.

The defining feature of the attached cultures is adherence to the substratum; thus it is not surprising that cell adhesion proteins are strongly expressed in the attached, but not in suspended cells, p-value 1.61E-8 (Table 2a). Eight integrin genes are differentially expressed, and so are additional keratinocyte transmembrane attachment proteins, ColXVII, Sarcoglycan, CD44 etc. Attached keratinocytes also produce their own ligands, the basement membrane proteins fibronectin, nidogen and laminins. Many of these proteins play important role in keratinocyte motility; consequently, genes that are positive regulators of motility are a prominently overexpressed category in the attached, not in the suspended keratinocytes (Table 2b).

**Table 2:** Ontological categories overexpressed in suspended keratinocytes.

a) Cell adhesion	b) Positive regulators of motility
ADAM metallopeptidase domain 9	ADAM metallopeptidase domain 9
CD44	SMAD family member 3
LIM and senescent cell antigen-like domains 1	SP100 nuclear antigen
actinin, alpha 1	actinin, alpha 1
catenin, beta 1, 88 kDa	actinin, alpha 4
collagen, type XVII, alpha 1	cAMP responsive element binding protein 3
connective tissue growth factor	cadherin 13, H-cadherin (heart)
fibronectin 1	chloride intracellular channel 4
integrin beta 1 binding protein 1	coagulation factor II (thrombin) receptor
integrin, alpha 2	coagulation factor II (thrombin) receptor-like 1
integrin, alpha 3	coagulation factor III (thromboplastin)
integrin, alpha 6	epidermal growth factor receptor
integrin, alpha V	histone deacetylase 9
integrin, beta 1	insulin receptor substrate 1
integrin, beta 4	insulin-like growth factor 1 receptor
integrin, beta 5	integrin, alpha 2
integrin, beta 6	integrin-linked kinase
integrin-linked kinase	gp130, oncostatin M receptor
laminin, alpha 5	jagged 1
laminin, beta 1	jagged 2
laminin, gamma 1 (formerly LAMB2)	laminin, alpha 3
nidogen 1	laminin, alpha 5
polycystic kidney disease 2	laminin, beta 1
protein tyrosine phosphatase, receptor type, K	matrix metallopeptidase 9
sarcoglycan, epsilon	mitogen-activated protein kinase kinase 1
thyroid hormone receptor interactor 6	neurofibromin 2 (merlin)
	platelet-derived growth factor alpha polypeptide
	podoplanin
	protein tyrosine phosphatase, receptor type, K
	related RAS (r-ras) oncogene
	related RAS (r-ras) oncogene2
	reticulon 4
	scavenger receptor class B, member 1
	serpin peptidase inhibitor, clade E (nexin)
	thrombospondin 1
	thyroid hormone receptor interactor 6
	transforming growth factor, beta 1
	tropomyosin 1 (alpha)
	v-akt oncogene homolog 1
	vascular endothelial growth factor C
	vinculin

a) Cell adhesion proteins. Note the many integrins in this category. b) Positive regulators of cell motility. Note the components of the extracellular matrix and basement membrane.

Interestingly, RNA splicing is also an essential regulated process in the attached keratinocytes, p-value 2.00E-16 (Table S5a). A large complement of HNRs and SNRs is overexpressed in the attached cells, as are the splicing factors, additional RNA binding proteins and Pol II components. This suggests that RNA processing, both mRNA and lncRNA [30]-[33], plays an important role in establishment or maintenance of basal, undifferentiated keratinocytes.

A key facet of the basal, proliferation-competent keratinocytes is energy supply. Consequently large number, 109, of mitochondrial proteins, including ATP synthase, cytochromes and mitochondrial ribosome proteins, are differentially overexpressed in the attached cells, p-value 4.45E-14 (Table S5b). Apparently, once started to differentiate, keratinocytes are no longer concerned with extensive energy supply.

We were surprised to find the ontological category ‘Melanosome’ prominently overexpressed in the basal cells, p-value 2.70E-12 (Table S5c). We note that we used pure cultures on keratinocytes, i.e., without melanocytes; thus the melanocyte-characteristic genes were not expected. A closer perusal of the genes in this category reveals that they do not include the melanin biosynthesis enzymes, such as Tyrosinase, Dopachrome tautomerase, Tyrosinase-related proteins 1 and 2, Silver or DHICA oxidase. Rather, the melanocyte-associated genes in the basal keratinocytes comprise transport and supporting genes, with functions related to endocytosis (e.g., annexins), chaperones (e.g., heat shock proteins), protein folding facilitators (e.g., isomerases), and membrane-resident auxiliary factors (e.g., stomatin, solute carriers etc.). These results suggest that basal keratinocytes are ‘primed’ to endocytose the melanosomes released from melanocytes and to process these melanosomes properly. If confirmed at the bench-top, the results suggest that melanosome-auxiliary factors are specifically made in the basal keratinocytes, and once the differentiation started the keratinocytes ‘passively’ transport melanin to the surface of the skin.

An important and very interesting feature of anoikis is its close relatedness to apoptosis [34]. Indeed, epidermal differentiation is sometimes compared to apoptosis, the parallels including DNA degradation, dissolution of internal organelles, protein crosslinking etc. [35], [36]. The mechanisms of anoikis and relatedly apoptosis in keratinocyte differentiation depends on signaling *via* integrin beta-1, and its inhibition is dependent on Bcl2 [37]–[39]. We found many apoptosis-related genes differentially overexpressed in the attached cells, p-value 2.06E-5 (Table S6). It is important to note that both positive and negative regulators of apoptosis are overexpressed and moreover that many genes in this category can be both pro- and anti-apoptotic (e.g., BCL2-associated X protein, Fas 6). These results, although difficult to interpret at the moment, perhaps suggest that basal keratinocyte are on one hand protected from apoptosis, on the other ‘primed’ to commence anoikis/apoptosis/differentiation once detached from the substratum.

### Metaanalysis of gene expression in suspended keratinocytes

Several agents have been suggested as inducers of keratinocyte differentiation *in vitro*, including confluence and inhibition of the JNK pathway [10], [17]. To compare the changes in transcriptomes due to such agents we used metaanalysis. We included in this analysis the transcriptional profiling of human epidermis *in vivo*, where the basal cells were separated from suprabasal based on the expression of the beta-4 integrin [18]. The full results of the metaanalysis are given in the supplement (Table S7; Table S8). In abbreviated form we present comparative analysis of genes induced by suspension in Table 3.

**Table 3:** Metaanalysis comparing the ontological categories overexpressed by inducers of keratinocyte differentiation.

Category cluster	GO Category	Suspended	Suprabasal	SP600125	Confluent	Conf-EGF
**Cornification**	∼ectoderm development	3.E-16	5.E-03	7.E-08	3.E-34	1.E-27
	∼epidermis development	3.E-15	2.E-03	1.E-07	9.E-33	1.E-28
	∼epidermal cell differentiation	2.E-12	1.E-02	7.E-04	4.E-29	1.E-21
	∼cornified envelope	4.E-13	2.E-04	2.E-04	1.E-23	1.E-16
**Innate immunity**	∼response to bacterium	1.E-03		8.E-03	5.E-02	7.E-02
	∼response to molecule of bacterial origin	8.E-03		1.E-03		
	∼response to lipopolysaccharide	1.E-02		4.E-03		
	∼inflammatory response	4.E-02		4.E-02		
**Lipid metabolism**	∼fatty acid metabolic process	3.E-02	4.E-03	3.E-02	5.E-02	8.E-02
	∼cellular lipid catabolic process	1.E-02		3.E-02	1.E-01	9.E-02
	∼lipid localization	4.E-03			1.E-02	6.E-02
	∼lipid transport	4.E-03			2.E-02	7.E-02
**Cholesterol metab.**	∼sterol biosynthetic process	9.E-02	3.E-03	2.E-03		
	∼steroid metabolic process	2.E-02	5.E-03	7.E-03		
	∼sterol metabolic process	6.E-02	3.E-03	3.E-02		
	∼response to steroid hormone stimulus	1.E-03		7.E-03		
**Apoptosis**	∼regulation of apoptosis	1.E-03	6.E-02	2.E-09		2.E-02
	∼regulation of programmed cell death	2.E-03	7.E-02	1.E-09		3.E-02
	∼regulation of cell death	2.E-03	7.E-02	2.E-09		3.E-02
	∼positive regulation of apoptosis	3.E-03		2.E-07		
**Organelles**	∼membrane-enclosed lumen	9.E-03	2.E-04	2.E-19		
	∼intracellular organelle lumen	6.E-03	6.E-04	2.E-19		
	∼organelle lumen	8.E-03	3.E-04	4.E-19		
	∼vacuole	8.E-06	5.E-04	8.E-03		6.E-03
**Transcription**	∼regulation of transcription RNA pol II promoter	1.E-08		2.E-10		
	∼transcription factor binding	2.E-06		1.E-11		
	∼chromatin	5.E-09		2.E-08		
	∼regulation of nucleoside, nucleotide metabolism	1.E-09		4.E-07		
**Proteolysis**	∼endopeptidase inhibitor activity	5.E-02	7.E-03	5.E-02	3.E-08	2.E-05
	∼peptidase inhibitor activity	7.E-02	1.E-02	8.E-02	6.E-08	4.E-05
	∼serine-type endopeptidase activity	2.E-02			7.E-08	1.E-05
	∼serine-type peptidase activity	1.E-02			2.E-07	2.E-05
**Protein kinase**	∼phosphorylation	7.E-06	4.E-02	5.E-06		
	∼protein amino acid phosphorylation	1.E-05		5.E-05		
	∼protein kinase activity	2.E-05		9.E-03		
	∼protein serine/threonine kinase activity	1.E-04		2.E-02		
**Protein phosphatase**	∼protein amino acid dephosphorylation	5.E-03	5.E-02			
	∼phosphoprotein phosphatase activity	1.E-02	2.E-02			
	∼phosphatase activity	9.E-03	3.E-02			
	∼dephosphorylation	8.E-03	6.E-02			
**Cell proliferation**	∼regulation of cell proliferation	3.E-04		1.E-10		
	∼negative regulation of cell proliferation	7.E-05		3.E-07		
	∼regulation of cell cycle	3.E-04		1.E-02		
	∼negative regulation of growth	2.E-02		6.E-02		
**Cell motility**	∼cell motion	2.E-03		3.E-04		
	∼cell migration	9.E-04		6.E-04		
	∼cell motility	2.E-03		2.E-03		
	∼localization of cell	2.E-03		2.E-03		
**Nucleus**	∼nuclear lumen	9.E-04	2.E-03	1.E-20		
	∼nucleoplasm	6.E-03	3.E-03	3.E-15		
	∼nucleoplasm part	1.E-02	2.E-02	1.E-11		
	∼nucleolus	9.E-02	5.E-02	3.E-07		
**Oxigen response**	∼response to oxidative stress	6.E-03		2.E-04	8.E-02	2.E-02
	∼response to reactive oxygen species	3.E-02		3.E-04	9.E-02	4.E-02
	∼response to hydrogen peroxide	5.E-02		2.E-04		
	∼response to hypoxia	5.E-03	6.E-02	5.E-02		

The p-values for each category are given for suspended, suprabasal, SP600125 JNK inhibitor treated and confluent keratinocytes in the absence or presence of EGF are given. Gray fields indicate that the given category did not reach statistical significance.

The most conspicuous ontological category, highly prominent in all transcription profiles, is the one of epidermal differentiation and cornified envelope components (Table 3). This category shows the highest p-values in suspended cultures.

Interestingly, suspended cultures and those treated with JNK inhibitor SP600125, induced components of innate immunity; these did not reach statistical significance in the suprabasal keratinocytes *in vivo*, or in confluent cultures. The enzymes of lipid metabolism, especially of fatty acids, are a component of epidermal differentiation process; they are turned on by all inducers of differentiation, but especially in suspension and in confluent cultures. In contrast, steroid metabolism enzymes, and coincidentally response to steroid hormones, are not induced by confluency; this aspect of epidermal differentiation, evident *in vivo*, seems specifically induced by detachment from the substratum and by the inhibition of the JNK pathway. Cytoplasmic organelle components, particularly of vacuoles and lysosomes, are also induced in differentiation promoters, although specifically not by confluency.

Suspension and JNK inhibition have several additional features in common, not shared by other inducers of differentiation; these include regulation of transcription, protein kinases, responses to oxidative stress, inhibitors of cell proliferation and cell motility. Nuclear proteins are very prominent in the transcriptomes of detached, JNK-inhibited and suprabasal cells; this is less so in confluent cultures. It is possible that these categories may be consequences of cell culturing, rather than intrinsic components of epidermal differentiation *in vivo*.

Interestingly, protein phosphatases are prominent aspects of differentiation *in vivo* and in detached keratinocytes. It is tempting to speculate that they play an important role in the detachment process itself. We note that proteolysis is a very prominent component of *in vivo* differentiation, shared by detachment from the substrate and by confluency. JNK inhibition seems less of an inducer of proteolysis.

When we compared the confluency-induced changes in keratinocytes grown in the presence or absence of EGF [17], we find virtually identical effects, except that apoptosis is induced in the absence, but not in the presence of EGF. This indicates that induction of apoptosis *per se* is not indispensable for epidermal differentiation. We note that the positive regulators of apoptosis are specifically induced in suspended cultures (in contrast, the negative regulators are induced in the attached cultures and the basal layer, see below).

### Metaanalysis of gene expression in attached keratinocytes

The most conspicuous ontological categories, highly prominent in all transcription profiles of not-differentiating cells, are the cell cycle and, related, the DNA replication machinery (Table 4). Similarly prominent are the interconnected categories of cell attachment to the substratum, basement membrane and extracellular matrix components. Interestingly, confluency by itself does not suppress these categories, they are as much a product of sub-confluent as of confluent cells. Indeed, the confluent cells remain firmly attached, while the suprabasal, detached, and JNK-inhibited cells stratify and thus lose bond with the substratum [10], [17], [18].

**Table 4:** Metaanalysis comparing the ontological categories overexpressed in undifferentiated keratinocytes.

Category Clusters	GO Categories	Attached	Basal	No SP600125	Sub-Conf.	Sub-C. -EGF
**Cell cycle**	∼cell cycle	9.E-19	2.E-03	2.E-07	1.E-21	2.E-30
	∼cell cycle process	2.E-18	7.E-04	1.E-06	1.E-21	1.E-28
	∼mitotic cell cycle	1.E-17	3.E-03	1.E-07	8.E-22	2.E-26
	∼cell cycle phase	1.E-12	1.E-02	5.E-06	1.E-21	1.E-31
**DNA replication**	∼DNA metabolic process	5.E-14	9.E-03	5.E-03	2.E-07	4.E-16
	∼DNA replication	5.E-08	5.E-05	4.E-03	1.E-05	6.E-09
	∼response to DNA damage stimulus	8.E-09	9.E-02	8.E-04	1.E-03	3.E-11
	∼DNA repair	1.E-07		5.E-02	5.E-03	3.E-11
**Cell attachment**	∼cell-substrate junction	1.E-07	7.E-07	7.E-02		1.E-04
	∼cell adhesion	1.E-04	3.E-05	5.E-06		6.E-05
	∼biological adhesion	1.E-04	3.E-05	5.E-06		6.E-05
	∼cell-substrate adhesion	2.E-06	9.E-05	7.E-04		5.E-03
**Basement**	∼basolateral plasma membrane	5.E-06	5.E-09	1.E-02		1.E-03
**membrane**	∼basement membrane	5.E-05	2.E-07	8.E-04		3.E-06
	∼basal lamina	1.E-02	2.E-04	4.E-02		3.E-03
	∼basal part of cell	4.E-03	2.E-02			3.E-02
**ECM**	∼extracellular matrix part	7.E-04	8.E-08	3.E-04		7.E-05
	∼extracellular matrix	2.E-02	1.E-06	4.E-03	2.E-02	5.E-05
	∼extracellular matrix binding	2.E-03	7.E-05	1.E-03		2.E-02
	∼collagen	9.E-02	5.E-04		3.E-02	6.E-02
**Organelle (ER)**	∼membrane-enclosed lumen	3.E-34	4.E-06	5.E-12	9.E-07	2.E-10
	∼organelle lumen	2.E-34	2.E-06	2.E-11	1.E-06	5.E-10
	∼intracellular organelle lumen	1.E-32	4.E-06	5.E-10	1.E-05	9.E-09
	∼endoplasmic reticulum	1.E-04	3.E-03	8.E-04	3.E-04	2.E-02
**Nucleus**	∼nuclear lumen	1.E-22	8.E-04	5.E-09	5.E-03	8.E-07
	∼nucleoplasm	2.E-16	3.E-04	4.E-08	4.E-04	4.E-07
	∼chromosomal part	6.E-08		4.E-04	6.E-11	7.E-15
	∼nucleolus	4.E-09	4.E-02	2.E-02		8.E-02
**Transcription**	∼chromosome	1.E-09		3.E-04	9.E-11	8.E-17
	∼response to vitamin	2.E-03	9.E-04	5.E-05	5.E-03	5.E-03
	∼negative regulation of gene expression	2.E-03	9.E-03	9.E-04		
	∼transcription factor binding	2.E-02	1.E-03	4.E-03		
**Splicing**	∼RNA splicing, via transesterification reactions	2.E-16	4.E-02			2.E-02
	∼RNA splicing, with bulged adenosine as nucleophile	2.E-16	4.E-02			2.E-02
	∼nuclear mRNA splicing, via spliceosome	2.E-16	4.E-02			2.E-02
	∼RNA binding	2.E-16				8.E-02
**Ribosome**	∼ribonucleoprotein complex	3.E-19				4.E-02
	∼protein complex assembly	3.E-10	6.E-03			4.E-03
	∼protein complex biogenesis	3.E-10	6.E-03			4.E-03
	∼ribosome biogenesis	1.E-06				
**Protein modif.**	∼negative regulation of protein metabolic process	3.E-08	2.E-03	3.E-02	4.E-02	3.E-04
	∼positive regulation of protein metabolic process	3.E-04	2.E-03	1.E-03	4.E-04	1.E-03
	∼negative regulation of protein modification process	7.E-07	9.E-04		5.E-03	2.E-04
	∼regulation of protein modification process	4.E-04	7.E-03	2.E-02	6.E-04	3.E-04
**Proteolysis**	∼regulation of ubiquitin-protein ligase during cell cycle	8.E-07	4.E-02		9.E-05	9.E-04
	∼regulation of ubiquitin-protein ligase activity	1.E-06	7.E-02		2.E-04	6.E-04
	∼regulation of protein ubiquitination	8.E-06	5.E-02	1.E-01	4.E-04	8.E-04
	∼positive regulation of ubiquitin-protein ligase activity	1.E-05	9.E-02		4.E-04	8.E-03
**Prot. Kinase**	∼intracellular signaling cascade	3.E-03	6.E-02	3.E-05	1.E-01	4.E-02
	∼kinase binding	1.E-04	4.E-03		5.E-02	3.E-03
	∼regulation of kinase activity	4.E-03		3.E-03	7.E-02	7.E-04
	∼regulation of protein kinase activity	9.E-03		4.E-03	6.E-02	2.E-03
**Cytoskeleton**	∼cytoskeleton	1.E-08	6.E-03	3.E-04	7.E-06	3.E-08
	∼cytoskeleton organization	2.E-08	3.E-04	1.E-02	1.E-03	7.E-10
	∼cytoskeletal part	4.E-05	9.E-02	2.E-02	7.E-08	9.E-07
	∼structural constituent of cytoskeleton	2.E-04	4.E-05			
**Actin/tubulin**	∼microtubule cytoskeleton	4.E-06		7.E-04	2.E-11	1.E-11
	∼actin cytoskeleton	1.E-07	4.E-03	7.E-02		
	∼microtubule cytoskeleton organization	2.E-05			8.E-05	3.E-08
	∼cytoskeletal protein binding	2.E-04	7.E-03	4.E-02	4.E-02	2.E-04
**Motility**	∼regulation of cell motion	6.E-07	2.E-04	8.E-04	2.E-02	5.E-04
	∼regulation of cell migration	4.E-07	4.E-04	3.E-03		5.E-03
	∼positive regulation of cell motion	2.E-06	2.E-02	6.E-03	1.E-02	2.E-03
	∼positive regulation of cell migration	9.E-07	1.E-02	2.E-02	7.E-02	2.E-03
**Mitochondria**	∼mitochondrion	4.E-14	5.E-05			7.E-03
	∼mitochondrial part	9.E-13	3.E-02			
	∼mitochondrion organization	6.E-07	4.E-03			
	∼generation of precursor metabolites and energy	8.E-09				
**Melanosome**	∼melanosome	3.E-12	1.E-03	4.E-07	7.E-02	
	∼pigment granule	3.E-12	1.E-03	4.E-07	7.E-02	
	∼pigment metabolic process	2.E-03				5.E-02
	∼pigment biosynthetic process	2.E-03				8.E-02
**Apoptosis**	∼regulation of cell death	1.E-05	1.E-03	5.E-06	2.E-02	6.E-02
	∼regulation of programmed cell death	3.E-05	1.E-03	4.E-06	2.E-02	6.E-02
	∼anti-apoptosis	2.E-03		4.E-07	4.E-02	
	∼negative regulation of cell death	9.E-05	7.E-02	2.E-05		

The p-values for each category are given for attached, basal, JNK inhibitor untreated and subconfluent keratinocytes in the absence or presence of EGF are given. Gray fields indicate that the given category did not reach statistical significance.

Organelle components are prominent in basal-like cells, but the organelles are those related to ER, in contrast with the suprabasal cells, which express vacuolar and lysosomal organelles. Principally, this suggests that the basal cells are more oriented toward synthesis, correct processing and secretion of new proteins, while the suspended ones are in a more catabolic, degradative mode.

As expected, nuclear processes and transcription in particular are important in maintaining the attached, basal state. Splicing and ribosome assembly are prominent in attached and basal cells and subconfluent cells in the absence of EGF, while they are less so in the presence of EGF. However, mitochondrial components are very prominent in the attached and basal cells; these do not change significantly due to JNK inhibition or in confluency. Interestingly, basal-like cells produce melanosomal components, as discussed above; the confluency does not seem to affect this process.

We note that the apoptosis regulators are prominent in all basal-like cells. However, unlike in the suprabasal-like, detached cells, it is the anti-apoptosis regulators that are expressed.

## Discussion

This study shows that detachment from the substratum initiates in keratinocytes a program of epidermal differentiation, which extensively, although imperfectly, parallels the *in vivo* differentiation program in skin. Specifically, detachment induces expression of cornified envelope components, desmosomal markers, innate immunity proteins, and lipid metabolism enzymes. Conversely, cell cycle and DNA replication, RNA splicing and transcription, adhesion, motility and extracellular matrix, and mitochondrial proteins are suppressed by detachment. In these aspects, the attached keratinocytes resemble the keratinocytes in the epidermal basal layer. Large assortment of transcriptional regulators is differentially expressed in suspended cells, accounting for the big changes in transcriptional profiles. The effects of detachment substantially overlap with those of other *in vitro* inducers of epidermal differentiation, such as confluency and inhibition of JNK.

Some transcriptional changes in suspended keratinocytes do not parallel those *in vivo*. For example, while suprabasal cells express many organelle components and protein metabolism enzymes, including proteases, suspended cells do not; conversely, suspended cells distinctively do express proliferation inhibitors, suprabasal cells do not.

The results are important because they comprehensively define the transcriptional changes caused by detachment of keratinocytes and establish that loss of attachment initiates a complex, many-sided program of epidermal differentiation. Thus, the results confirm and substantially expand the original observations by the Watt group that detachment induces involucrin synthesis [11], [12]. Furthermore, detached cells represent a very useful model for studies of differential responses of basal *vs.* differentiation keratinocytes to cytokines and other extracellular signals. For example, IL-17, a very important effector in psoriasis [40], was shown to act to a significant extent via C/EBPb, transcription factor characteristic of differentiating cells [41]. This model might prove useful for studies of cutaneous HPV, a notoriously difficult experimental problem [42].

The mechanism of detachment-induced differentiation presumably involves integrins, which have been identified as the sensors of detachment and initiators of differentiation [14]. Indeed, we find that many integrin genes, expressed in the attached cells, are not expressed in suspension. The signaling pathway responsive to integrins involves G-proteins, interactions with membrane receptors and protein kinases [13], [43], [44]. Indeed, we find that many MAPKs are induced by detachment. However, integrins may not be the only signal for differentiation because confluency in culture, which also initiates various aspects of differentiation, does not change substantially the range of integrins, basement membrane proteins and other attachment factors. The most consistent process associated with differentiation is cessation of DNA replication and cell cycle; all pro-differentiation agents counter the cell cycle. Therefore it is tempting to speculate that, while there is more than one way to initiate differentiation, they all require the blocking of the cell cycle.

On the other hand, blocking the cell cycle is not sufficient for all components of the differentiation to commence; specifically, confluency induces fatty acid metabolic enzymes, but not the cholesterol metabolic ones. This is not simply a consequence of *in vitro* growth conditions, because detachment and JNK inhibitors induce the metabolic enzymes of both. Therefore, it appears that individual components of the differentiation process, such as innate immunity, steroid metabolism etc. while coordinated *in vivo*, are on separate, individual regulatory branches, i.e., can be controlled individually.

It is important to recognize the limitations of the methods used in this study. Specifically, it is possible that several ontological categories are important in the processes described, but do not meet the statistical significance cut-offs chosen. For example, confluency may induce fewer innate immunity genes, or induce them to a lesser degree, which would exclude these from Table 3. Apoptosis regulators can often be both positive and negative and their transcriptional changes do not necessarily imply the presence or absence of apoptosis. Despite such caveats, it is clear that detachment from the substratum initiates a quite comprehensive differentiation process.

## Materials and Methods

### Keratinocyte growth in suspension

We used the approach described before [45], [46]. Briefly, primary cultures of normal human neonatal foreskin epidermal keratinocytes were obtained from Dr. M. Simon (Living Skin Bank, Burn Unit SUNY Stony Brook). The cultures were initiated using 3T3 feeder layers and then frozen in liquid nitrogen until used. Once thawed, the keratinocytes were grown without feeder cells in a defined serum-free keratinocyte growth medium, KGM, supplemented with 2.5 ng/ml epidermal growth factor and 0.05 mg/ml bovine pituitary extract (keratinocyte-SFM, GIBCO, San Diego, CA) at 37°C, in 5% CO_2_. The medium was replaced every 2 days and the cells were expanded through 3 passages for the experiments. They were trypsinized with 0.025% trypsin, which was neutralized with 0.5 mg/ml of trypsin inhibitor. We avoid using serum to neutralize the trypsin because serum can promote certain aspects of keratinocyte differentiation. For most of our experiments, we use third-passage keratinocytes at 50-70% confluence. In this work we used the same, single line of primary human keratinocytes described in our previous publications [6], [10], [45], [46]. These primary cultures provide a more appropriate target than immortalized, anneuploid cell lines, and by using a single large batch of cells, we avoided variability due to growth conditions. Suspended keratinocytes were plated into bacteriological, not pre-treated Petri dishes, where they fail to attach to the substrate. They were harvested by centrifugation. Attached keratinocytes were grown in standard P100 tissue culture dishes and the cells were harvested by scraping.

### RNA isolation and labeling

To obtain RNA of appropriate quality for chip analysis we first disrupted the keratinocytes and isolated the RNA is using Trizol (Gibco). This was followed by the use of Qiashredders to homogenize cell extracts with centrifugation at 1,800×g for 2 min. The DNA was removed with on-column DNAse digestion using RNAses-free DNAse Set (Qiagen). The RNA samples were stored in water at −80°C until hybridization. To ascertain the good quality of RNA, 28S and 18S ribosomal bands are visualized on a non-denaturing agarose gel and OD_260/280_ spectrophotometric ratio of at least 1.8 was determined. Approximately 5 to 8 µg of total RNA was reverse transcribed, amplified and labeled as described [6], [47]. Labeled cRNA was hybridized to the arrays (Affymetrix U95Av2), which were washed, stained with anti-biotin streptavidin-phycoerythrin labeled antibody using Affymetrix fluidics station and then washed again according to the Affymetrix protocol. The arrays were scanned using the Agilent GeneArray Scanner system (Hewlett-Packard) and GeneChip 3.0 software to determine the expression of each gene. The Northern blot analyses that confirm microarray data used standard molecular biology protocols, as described [45], [46]. The Affymetrix U95Av2 arrays contain 12,625 features and probe almost 10,000 full-length human genes.

### Array data analysis

We used the same data analysis approach as described in our publications [48]. Intensity values from the chips were obtained using Microarray Suite v. 5.0 (Affymetrix), and scaled by calculating the overall signal for each array. We included in the analysis only those genes determined by the Affymetrix algorithm to be present in at least one sample. The eliminated genes are not expressed in keratinocytes, or expressed at such low level that their measurements were unreliable. Differential expression of transcripts was determined by calculating the fold change, where genes were considered regulated if the expression levels differed more than 1.5-fold. Annotation and ontology of regulated genes was obtained using DAVID [49].

Microarray data were deposited into annotated and curated database of the National Center for Biotechnology Information (NCBI) Gene Expression Omnibus, GEO (http://ncbi.nlm.nih.gov/geo) [50], and are accessible under GSE57045.

## Supporting Information

Table S1 **Genes overexpressed in suspended keratinocytes.**
(XLSX)

Table S2 **Genes overexpressed in attached keratinocytes.**
(XLSX)

Table S3 **Positive and negative regulators of transcription overexpressed in suspended keratinocytes.**
(XLSX)

Table S4 **Cell cycle and proliferation associated overexpressed in attached keratinocytes.**
(XLSX)

Table S5 **Splicing, melanosomal and mitochondrial genes overexpressed in attached keratinocytes.**
(XLSX)

Table S6 **Apoptosis regulators overexpressed in attached keratinocytes.** A few genes that can serve as both positive and negative regulators are marked in gray, as an illustration.
(XLSX)

Table S7 **Complete results of metaanalysis comparing ontological categories in differentiating keratinocytes.** Gray field indicate p-values above 0.1.
(XLSX)

Table S8 **Complete results of metaanalysis comparing ontological categories in basal, undifferentiated keratinocytes.**
(XLSX)
